# Polysaccharide-Bacteria Interactions From the Lens of Evolutionary Ecology

**DOI:** 10.3389/fmicb.2021.705082

**Published:** 2021-10-08

**Authors:** Andreas Sichert, Otto X. Cordero

**Affiliations:** Ralph M. Parsons Laboratory for Environmental Science and Engineering, Department of Civil and Environmental Engineering, Massachusetts Institute of Technology, Cambridge, MA, United States

**Keywords:** polysaccharides, biodegradation, microbial cooperation, microbial ecology, population dynamics, microbial communities, bacterial evolution

## Abstract

Microbes have the unique ability to break down the complex polysaccharides that make up the bulk of organic matter, initiating a cascade of events that leads to their recycling. Traditionally, the rate of organic matter degradation is perceived to be limited by the chemical and physical structure of polymers. Recent advances in microbial ecology, however, suggest that polysaccharide persistence can result from non-linear growth dynamics created by the coexistence of alternate degradation strategies, metabolic roles as well as by ecological interactions between microbes. This complex “landscape” of degradation strategies and interspecific interactions present in natural microbial communities appears to be far from evolutionarily stable, as frequent gene gain and loss reshape enzymatic repertoires and metabolic roles. In this perspective, we discuss six challenges at the heart of this problem, ranging from the evolution of genetic repertoires, phenotypic heterogeneity in clonal populations, the development of a trait-based ecology, and the impact of metabolic interactions and microbial cooperation on degradation rates. We aim to reframe some of the key questions in the study of polysaccharide-bacteria interactions in the context of eco-evolutionary dynamics, highlighting possible research directions that, if pursued, would advance our understanding of polysaccharide degraders at the interface between biochemistry, ecology and evolution.

## Introduction

The degradation of complex organic matter by microbes is a key process in the global carbon cycle. The bulk of organic matter in soils and oceans is in the form complex and high-molecular organic matter such as proteins, aromatic compounds and polysaccharides (Benner et al., 1992; Kögel-Knabner, 2002), which constantly undergo recycling by microbes controlling the ecosystem's carbon reservoir. To initiate the recycling of these materials, microorganisms like bacteria synthesize complex enzymatic machineries that break down the polymeric substrates, triggering a cascade of metabolic conversions that ends with the remineralization of organic matter back to its inorganic sources.

Bacterial pathways for polysaccharide degradation are elaborate multi-step reactions with highly substrate-specific enzymes. Microbial enzymes “read” the structural information of polysaccharides by accommodating specific carbohydrate structures in substrate binding sites (Henrissat and Davies, 1997). Indeed, carbohydrate active enzyme (CAZymes) are highly substrate specific to single structural motifs, and many new substrate specificities are still being discovered (Lombard et al., 2014; Tamura and Brumer, 2021). The CAZymes that break down a target polysaccharide are often co-located and regulated in polysaccharide utilization loci (PUL) (Martens et al., 2008). PULs are highly specific for their target polysaccharide and encode the machinery to sense, bind, depolymerize, and take up polysaccharides (Martens et al., 2009; McKee et al., 2021). While structurally simple polysaccharides such as starch or laminarin can effectively be degraded by three or four enzymes encoded in small PULs (Martens et al., 2009; Unfried et al., 2018; Becker et al., 2020), complex branched polysaccharides such as ulvan or pectin require a cascade of up to 21 debranching enzymes each acting in correct sequence prior to full access to the polysaccharide backbone (Ndeh et al., 2017; Reisky et al., 2019). The number of required enzymes jumps to the hundreds in the case of the highly branched and sulfated polysaccharide fucoidan from brown algae (Sichert et al., 2020). These examples suggest a direct mapping between the complexity of polysaccharides and degradation pathways.

In recent years, genomic data has revealed that the pathways for polysaccharide degradation evolve rapidly, changing over few generations through rampant gene gain (via horizontal gene transfer, HGT) and loss. This fast evolution has the potential to change strategies used by bacteria to degrade polysaccharides, as well as the interactions with neighboring community members (Hehemann et al., 2016). Such interactions have been postulated to drive the overall dynamics of degradation (Lehmann et al., 2020), which is why the stability of organic matter is frequently described as an emergent property of the underlying microbial communities (Schmidt et al., 2011; Dittmar et al., 2021). Thus, gaining a more complete understanding of the processes that modulate the breakdown of complex organic matter by microorganisms may require us to consider, not only the biochemistry of hydrolytic pathways, but the ecological and evolutionary processes that shape the diversity of degradation strategies and enzyme repertoires. In this perspective we take a stab at this problem by discussing a few of the most pressing, open challenges in the study of polysaccharide-bacteria interactions from the viewpoint of evolutionary ecology (Box 1).

Box 1 **Six conceptual challenges in polysaccharide degrading communities.**

1. **Mapping of polysaccharide complexity to genetic repertoires**
  Microbial pathways evolve to match polysaccharide structure, yet it is unclear how PULs evolve in response to subtle changes in the structure of the substrate, e.g., PULs can either be progressively updated or alternative degradation routes can evolve.
2. **Adaptive vs. non-adaptive evolution of enzyme repertoires**
  Why do CAZyme repertoires evolve so rapidly? Answering this question requires us to understand the fitness landscape of polysaccharide degradation pathways, its structure (i.e., ruggedness) and dynamicity.
3. **Sensing, regulation and phenotypic heterogeneity**
  Intermediate oligosaccharides act as signals that regulate the metabolism and behavior of degraders. Yet, the full range of responses to these signals and the role of single cell heterogeneity remains underexplored.
4. **Trait based ecology**
  Polysaccharide degraders can be described in terms of key traits, such as substrate affinity, hydrolysis rates and maximal growth rate. Trade-offs in trait space may lead to the evolution of alternative degradation strategies. Describing degraders in terms of traits, and not taxonomy, is a major challenge in the field.
5. **Impact of microbial interactions on hydrolysis rates**
  In the environment, bacteria form distributed metabolic systems connected by the exchange of metabolites, yet the effect of these interactions on overall degradation in natural environments remain largely unclear.
6. **Non-linear growth kinetics**
  Bacterial growth on polysaccharides can be density dependent, such that degradation only takes place at high local bacterial densities. It is unclear to what extent polymer recalcitrance in the environment stems from this form of non-linear growth kinetics.

## Challenge 1 | Mapping of Polysaccharide Complexity to Genetic Repertoires

In-depth biochemical characterizations of PULs have revealed a tight mapping between PUL enzyme content and target polysaccharide structure: one enzyme is required per unique glycosidic linkage, accompanied by endo-acting enzymes that initiate depolymerization (Ndeh et al., 2017; Reisky et al., 2019). In recent years, PULs for many important polysaccharides have been described including mannan, xylans, n-glycans, carrageenan or ulvan (Cuskin et al., 2015; Rogowski et al., 2015; Ficko-Blean et al., 2017; Briliute et al., 2019; Reisky et al., 2019). Yet, many more await characterization. The number of PULs discovered using SusD as a marker gene in *Bacteroidetes* appears to saturate to a few thousand, suggesting that a biochemical study of PUL diversity in the human gut as well as marine environment is tractable (Krüger et al., 2019; Lapébie et al., 2019). Linking these PULs to their substrates and revealing their different degradation pathways should give us a unique perspective of how primary degraders of organic matter and polysaccharides have coevolved.

**Figure 1:**
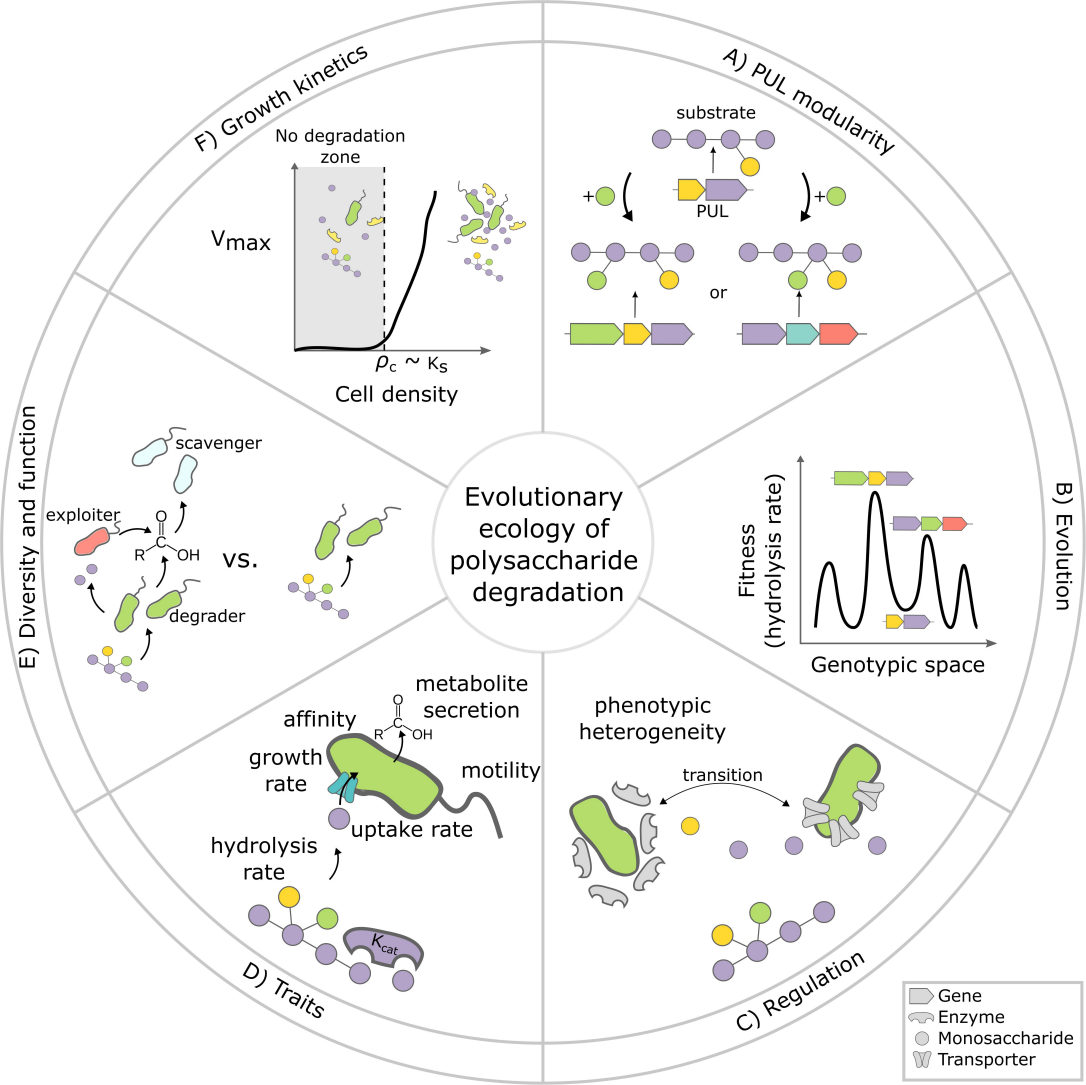
Evolutionary ecology of polysaccharide degradation: six key challenges. **(A)** What determines whether a PUL evolves by incremental updates? What type of polysaccharide modifications (e.g., green monosaccharide) require a large jump in genotype space (i.e., complete replacement of the PUL) vs. simple updates? **(B)** How rugged and dynamic is the fitness landscape of PULs? The illustration shows a 1D-projection of a hypothetical n-dimensional PUL fitness landscape. PULs can evolve by mutation, gene loss or gain (x-axis), which changes the PUL's hydrolysis rate, which we assume here to be equivalent to fitness (y-axis). Local fitness minima (ruggedness) can constrain the evolutionary trajectories of PULs, promoting their diversification. **(C)** How widespread is phenotypic heterogeneity and division of labor among degraders? For complex polysaccharides, only a small subpopulation of cells expresses enzymes, while others benefit from released hydrolysis product without expressing enzymes (adapted from Tatli et al., 2021). Intermediates such as mono- and oligosaccharides might regulate the transition between specialized subpopulations. **(D)** What are the key physiological parameters (i.e., traits) that define the ability of bacteria to compete for polysaccharide degradation, and how are these traits quantitatively related to one another? Likely candidate traits are the affinity to monomers, hydrolysis rates or the ability to swim toward breakdown products. **(E)** What is the impact of species and strain diversity on the function of polysaccharide degrading bacteria? In the environment, polysaccharide degrading communities are often dominated by bacteria that do not directly contribute to degradation such as exploiters and scavengers (red and turquoise cells), but feed on metabolites released from degraders (green cells). The impact of these non-degraders on overall degradation rate is largely unknown. **(F)** Is apparent recalcitrance a consequence of non-linear growth kinetics? Critical cell density thresholds, which emerge due to diffusive loses of breakdown products, imply that polysaccharides are recalcitrant when cell numbers are low. How low the threshold is, depends on physiological parameters like the half saturation constant of the Monod uptake kinetics for monomers (*Ks*). Cell-cell aggregation is a facultative strategy to deal with cell-density thresholds.

However, despite its appeal, this one “PUL – one substrate” paradigm may fall short. Consider the question of how small changes in the polysaccharide structure drive the enzyme content of PULs. As the structure of plant polysaccharides such as xylans or pectins is largely conserved between plant species, their structure only varies in subtle side chain modifications (Fangel et al., 2012). To handle such ramified structures, bacterial evolution can follow one of two strategies: (a) acquire a completely new PUL, performing a distinct degradation pathway, or (b) “upgrade” their existing PUL by acquiring the enzymes needed to remove side chain modifications, while conserving the breakdown pathway (Figure 1A). An example of the former strategy is observed in microorganisms that degrade xylans with additional backbone modifications, which require a different PUL than the one that targets unmodified xylans (Rogowski et al., 2015). By contrast, the latter evolutionary strategy is observed in organisms that degrade branched xyloglucans, whose different branches are systematically trimmed and subsequently digested by a core pathway (Larsbrink et al., 2014). To the extent that PULs can be progressively upgraded to deal with polysaccharide modifications, evolution should proceed in small steps. Otherwise, a small change in polysaccharide structures may lead to complete loss of homology, i.e., by using an alternative set of enzymes required to degrade heavily decorated substrates (Razeq et al., 2018). It will be interesting to learn what modifications trigger such drastic changes in pathway structure vs. more incremental solutions.

Related to this problem is the question of to what extent alternative degradation strategies are used to digest the same polysaccharide. Theoretically, complex polysaccharides have multiple alternative degradation routes, which can depend on the order and position of enzymatic cleavage. For example, chitin [a polymer of (1→ 4)-β-linked N-acetyl-D-glucosamine] can either be deacetylated first, yielding chitosan, and then depolymerized or vice versa (Beier and Bertilsson, 2013). Interestingly, the involved endo-enzymes in chitin or chitosan degradation are selective for the presence or absence of N-acetyl (Saito et al., 1999; Malecki et al., 2014), which suggest that both pathways are mutually exclusive and may not be operated simultaneously within the same organism. Furthermore, there are well-known examples of convergent CAZyme evolution, for example, different endo-glucanases independently evolved from structurally different ancestors to cleave the same polysaccharide (Lombard et al., 2014). These alternative CAZymes create possibilities to assemble alternative pathways for the same polysaccharide by substituting one required function with a functional homolog. For example, to hydrolyze beta-glucans, Bacteroidetes use GH16 endo-glucanases in combination with GH30, GH17 or GH3 (Becker et al., 2017; Tamura et al., 2017), whereas certain Firmicutes use GH161 endo-glucanases in combination with GH1 or GH94 (Kuhaudomlarp et al., 2019). The extent to which alternative functional homologs can impact yield, efficiency or produced oligosaccharides is unknown. Exploring these alternative pathways in microbial genomes and characterizing them in terms of physiological traits (see below), would represent a significant step toward better understanding the evolution of polysaccharide degraders.

## Challenge 2 | Adaptive vs. Non-adaptive Evolution of Enzyme Repertoires

Polysaccharide degrading organisms often feature an expanded repertoire of seemingly redundant CAZymes (Hehemann et al., 2016; Enke et al., 2018; Sichert et al., 2020). Similarly, microbial ecosystems often contain multiple primary degraders for the same substrate with varying number of enzyme homologs (Vanwonterghem et al., 2016; Woodcroft et al., 2018). The factors that drive this apparent redundancy are generally unclear, but two opposing perspectives arise:

One perspective is that genetic repertoires are tightly “optimized” by evolution, such that every enzyme has a specific function. Consequently, the more enzymes an organism carries the better it can perform the task of breaking down the substrate. This view is supported by recent studies: In a collection of marine Vibrios capable of degrading alginate, CAZyme copy number correlated with degradation rates and preferred size of alginate, allowing us to infer these phenotypic characteristics from genomes (Hehemann et al., 2016). Similarly, degraders of chitin in the ocean typically contain multiple chitinases, up to 19 in bacteria that colonize chitin particles (Enke et al., 2018), despite the fact that only three chitinases are needed to break down chitin (Beier and Bertilsson, 2013). On the surface, these studies support the reductionist notion that degradation rates can be understood by focusing solely on hydrolase repertoires. However, degradation rates could be affected by many other traits, such as the ability to swim toward breakdown products and uptake them into the cell, among others (Pollak et al., 2021). To what extent the ecological success of a degrader and the size of its hydrolase repertoire are directly or indirectly linked, remains unclear, but this is a tractable a problem that can be addressed with genetic model systems such as vibrios (Visick et al., 2018) or *Z. galactanivorans* (Zhu et al., 2017).

Another less obvious perspective is that the features that we can observe in sequenced genomes are not necessarily fine-tuned by evolution, but snapshots of a dynamic process that can generate non-adaptive variation as populations navigate complex fitness landscapes. This is likely, because CAZyme repertoires evolve rapidly by frequent horizontal gene transfer and gene loss, facilitated by mobile genetic elements such as integrative conjugative element and plasmids (Koch et al., 2020; Pudlo et al., 2020). This gain and loss of CAZymes is in fact so rapid that closely related bacteria can often differ significantly in their PULs (Hehemann et al., 2012, 2016). This mode of evolution suggests a constant search for new genetic variants as opposed to conserving existing ones, indicating that the underlying fitness landscape of PULs is rugged and / or dynamic. Rugged fitness landscapes result from epistatic interactions (de Visser and Krug, 2014), such as those emerging from incompatible enzymatic pathways, which render certain configurations of the enzymatic repertoire deleterious for the organism (Figure 1B). Imagine a simple scenario where a bacterium imports a specific oligosaccharide for periplasmatic breakdown; acquiring an extracellular enzyme that degrades this oligosaccharide would result in lower efficiency of the transporter and reduce the bacterium's fitness. These effects can render certain enzyme combinations deleterious and favor organisms that can rapidly recombine and jump large distances in genotypic space.

Dynamic fitness landscapes are those where the structure of the landscape is highly dependent on the environment, including biotic factors such as population size or density. One process that can create this dynamicity is social cheating. Social interactions have been shown to be a relevant process in polysaccharide degrading marine and gut microbes, leading to loss-of-function mutants that exploit the hydrolysis byproducts released by degraders (Pollak et al., 2021). This form of social interaction can turn a highly fit degrader, with an optimal breakdown pathway, unfit in an environment with high population density (Gore et al., 2009). Therefore, evolving into a highly specialized degrader can be highly advantageous, but only in a narrow space of parameters. This tension between selective pressures to expand and contract the enzymatic repertoire can explain, in part, the large variability of hydrolase content between close relatives. Moreover, being able to quickly diversify the enzyme content in order to adapt to ever changing environments at fast rates is also likely advantageous, possibly explaining why hydrolases and PULs are often linked to mobile genetic elements.

## Challenge 3 | Sensing, Regulation, and Phenotypic Heterogeneity

Different lines of evidence indicate that byproducts of oligosaccharide breakdown can act as signals, that regulate not only the transcription of PULs, but a large range of cellular behaviors. In the genomes of degraders, the periplasmatic stimulus binding domain of histidine kinases differentiates between structurally similar oligosaccharides, with sufficient structural information to only induce the target PUL (Martens et al., 2011). These stimulus oligosaccharides are often generated by constitutively expressed endo-acting CAZymes referred to as sentry enzymes (Thomas et al., 2012), which can also be directly involved in sensing structurally different polysaccharides (Sonnenburg et al., 2010). Importantly, intermediate break-down products can also be sensed by non-degrading bacteria, inducing dramatic changes in behavior. For example, mucin derived oligosaccharide can inhibit virulence in *P. aeruginosa* (Wheeler et al., 2019), whereas free fucose cleaved from mucin increase virulence of *E. coli* (Pacheco et al., 2012). Also, motility can be regulated by cyanobacterial mucin and derived oligosaccharides (Reddi et al., 2018) or xylose in *Caulobacter crescentus* (D'Souza et al., 2021). However, there is still much to learn about the potential for oligosaccharides to act as control “knobs” of bacterial behavior. For instance, it's unclear how microbes respond to oligosaccharide combinations, if those responses are additive or if signals interact. Given the large number of possible combinations, this is an avenue that could be explored as a potential tool for future microbiome interventions.

Another important aspect is the fidelity with which organisms respond to signals like oligosaccharides. In many scenarios, different cells of a clonal population in the same environment can diverge in their gene expression patterns. In the case of organisms that degrade complex polysaccharides, this divergence may serve an important role, given the significant investment of cellular resources required to express enzymatic repertoires. For example, only a subset of 20–80% of *C. thermocellum* produces cellulosomes, whereas the remainder of the population benefits from released hydrolysis product (Tatli et al., 2021). This division of labor may be “optimal” for population growth, as cellulosomes are large macromolecular structures that encase cells and likely slow down cell division (Figure 1C). In general, the expression of large enzymatic repertoires can impede growth, as less resources are allocated to biomass production (Basan et al., 2015). Therefore, we expect phenotypic heterogeneity to play a significant role in populations carrying large enzyme repertoires, such as the Verrucomicrobia that target fucoidan (Sichert et al., 2020) or Bacteroidetes that need to coordinate large number of PULs (Klassen et al., 2021a). Overall, the potential benefits of these forms of phenotypic heterogeneity represent an exciting frontier in the study of polysaccharide-bacteria interactions.

Catabolite repression regulates the substrate preferences of polysaccharide degraders, which could also impact the degradation rates of microbial communities. In pure cultures, catabolite repression regulates the prioritization of certain polysaccharides over others (Tuncil et al., 2017; Koch et al., 2019), which was also observed in bacteria that colonize particles (Bunse et al., 2021). These substrate preferences could be particularly important in natural environments, where microbial communities are exposed to mixtures of polysaccharides and catabolite repression could govern their sequential hydrolysis. For example, during a diatom spring bloom, simple substrates such as laminarin and starch were used throughout the bloom, whereas more complex cell wall polysaccharides were used in the later stages of the bloom (Francis et al., 2021; Vidal-Melgosa et al., 2021). This shows that microbial ecosystems display a hierarchy of substrate utilization, yet it is unclear if these patterns are caused by gene regulation or alternatively, by a succession of different specialized degraders within the microbial community (Teeling et al., 2012). Exploring how microbes regulate their enzyme repertoire in the environment and identifying the molecular regulatory mechanism that could control substrate preferences are at the heart of microbial ecology and would help to understand the ecosystem-level regulation of polysaccharide degradation.

## Challenge 4 | Trait Based Ecology

A trait can be any measurable characteristic that contributes to the organisms' fitness. Trait-based ecology describes communities, not in terms of species collections, but as collections of individuals occupying a trait space. Ecologically relevant “traits” for polysaccharide degraders could be for example the growth rate on a polysaccharide, the rate of enzyme secretion, the uptake rate of mono- and oligomers or carbon use efficiency (Allison, 2012). Such a trait-based description of a polysaccharide degrader would allow us to focus on the key physiological parameters that contribute to the organism's competitive ability, independently of molecular and taxonomic details (Figure 1D). Moreover, the importance of measuring these key physiological parameters lies in the discovery of “trade-offs,” or anticorrelations between traits, that reveal fundamental metabolic or physiological limitations that constrain evolution. Examples of trade-offs could be growth rate vs. biomass yield (Pfeiffer et al., 2001; Molenaar et al., 2009), enzyme secretion rate vs. monomer affinity or enzyme privatization vs. hydrolysis rate.

Trade-offs such as these, or their combination, could explain the large diversity of degraders that can coexist in the environment, and even in simple laboratory enrichments. For instance, while many degraders use external hydrolysis with secreted enzymes and monomer transporters, some degraders use membrane-tethered endo-acting enzymes that produce oligosaccharides taken up by specialized transporters in a “selfish” manner, i.e., they release no detectable extracellular monosaccharides (Cuskin et al., 2015; Reintjes et al., 2017). It is likely that the coexistence of these two modes of extracellular and selfish strategies results from a fitness trade-off that favors selfish uptake at low carbon concentration and external hydrolysis at high carbon concentrations (Arnosti et al., 2021).

However, although trade-offs can be easily postulated and plugged into mathematical models, the challenge is fundamentally empirical as most if not all of these trade-offs lack proper experimental evidence. This is because of serious technical—as well as conceptual—limitations to measure key traits. For instance, one of the key traits in any model of bacterial growth and competition is the effective affinity of cells to the substrate, typically referred to as *Ks*, and equal to the inverse of the half-saturation constant of the Monod uptake kinetics. Measurement of this key parameter often requires growing organisms in a chemostat, which is hard to do in sufficient high-throughput to generate a meaningful picture of the trait space. Yet, the advance of cultivation independent methods to capture microbial physiology (such as the uptake of fluorescently-labeled polysaccharides by selfish bacteria) provides an exciting avenue to measure microbial traits (Hatzenpichler et al., 2020; Klassen et al., 2021b). While the development of a general trait-based ecology for microbes may be unrealistic given the large diversity of microbial metabolisms, it is reasonable to hope that a trait-based approach can be developed for the specific case of polysaccharide degradation, given that many of the key traits that affect degradation and growth rates are known. This is an exciting frontier at the interface between physiology, evolutionary biology and mathematical modeling.

## Challenge 5 | Impact of Microbial Interactions on Hydrolysis Rates

Some polysaccharide degraders are “sloppy feeders,” releasing hydrolysis byproducts and metabolic waste that can promote the growth of secondary consumers (Paczia et al., 2012; Wienhausen et al., 2017; Ebrahimi et al., 2019; Gralka et al., 2020). For example, oligosaccharide exploiters, which grow on hydrolysis products but do produce hydrolases (a.k.a “cheaters”), have been shown to invade chitin degrading communities in the ocean, competing for carbon with degraders and reducing their yield (Pollak et al., 2021). Both degraders and exploiters release amino acids and TCA cycle intermediates such as malate or succinate (Enke et al., 2019; Pontrelli et al., 2021) that fuel the growth of scavengers specialized on consuming these metabolites. Likewise, during fucoidan degradation, fucose is partially respired and fermented into large amounts of lactate and propanediol (Sichert et al., 2020), which is likely consumed by neighboring microbes. These processes mediate the assembly of diverse communities in which primary degraders may only represent a small fraction. A key challenge in the field is to understand the impact that non-degrading community members on the degradation kinetic and the fate of carbon (Figure 1E).

Although the problem remains open, studies within marine chitin degrading communities (Datta et al., 2016; Enke et al., 2018) suggest that the presence of non-degrading organisms in the community decreases degradation rates and total productivity. The mechanisms mediating these effects are generally unknown, however, it is reasonable to hypothesize that the decrease in degradation results from competition for space on particle surfaces, essential nutrients, or oligosaccharides in the specific case of cheaters. The negative impact of secondary consumers on degradation observed in these marine systems is in stark contrast with the well-known case of end-product inhibition in anaerobic environments, where fermentation products such as hydrogen can be inhibitory due to the low Gibbs free energy of the reaction (Iyer and Lee, 1999). The removal of hydrogen by hydrogenotrophic methanogens allows the reaction to move forward, thus creating a *de facto* mutualistic interaction between fermenter and methanogen (Sasaki et al., 2012).

Beneficial interactions between microbes are more likely to occur when the environment is limiting for multiple nutrients, such as iron or vitamins. In such limiting environments, it may be possible for degrading and non-degrading populations to engage in mutualisms that enhance degradation, for instance if non-degraders contribute with public goods such as iron-scavenging siderophores. However, further work is needed to understand the interplay between abiotic conditions and community composition, as well as its impact on polysaccharide degradation. This is an important challenge because polysaccharide degraders never act alone, but in the context of diverse consortia.

## Challenge 6 | Non-linear Growth Kinetics

Although a polymer may be intrinsically degradable, it can appear as recalcitrant in the environment due to the non-linear growth kinetics of bacteria. Modeling shows that the key trait that controls this phenomenon is *Ks*, the affinity of cells to degradation byproducts (Ebrahimi et al., 2019). If *Ks* is equal or larger than the monomer concentration experienced by cells, the population may not be able to grow sufficiently fast to overcome natural mortality or dilution (Figure 1F). This is a manifestation of what is known as an Allee effect in population ecology (Rosenberg et al., 1977; Lewis and Kareiva, 1993). For example, the marine microbe *Psychromonas* sp. 6C06 can only degrade chitin when in high cell numbers, 10^4^ cells/particle (Ebrahimi et al., 2019), which is high relative to an average 10^6^ cells/mL in seawater. Incidentally, when its cell density is above the critical threshold, this organism can degrade chitin faster than any other member of the community from which it was isolated (Enke et al., 2018). This positive density dependent, or cooperative, growth, can be particularly important in aqueous, dilute environments like oceans or lakes, where degradation can be bacteria-limited. Although models show that cooperative growth kinetics can emerge without the need for regulation (Ebrahimi et al., 2019), further work should test to what extent mechanisms that sense local density, such as quorum sensing, control the expression of complex enzyme repertoires in the environment. Overall, the existence of cell density thresholds illustrate how recalcitrance can be an emergent property of the interaction between polymers and bacteria, and not necessarily an intrinsic feature of polysaccharides.

Positive density dependent growth also provides us with a mechanism to explain so-called priming effects (Fontaine et al., 2003). This is because the addition of labile substrates could increase the population size of degraders, thereby enabling the recycling of more recalcitrant forms of organic matter. The implications of this hypothesis were recently explored using consumer – resource models, which can qualitatively reproduce the distribution and concentration of organic matter in the ocean (Mentges et al., 2019; Zakem et al., 2021). The underlying key assumption was that most substrates are accessible to generalists and few substrates only to specialists. This resulted in high cell numbers of generalists and low cell numbers of specialized degraders, suggesting that the interplay between specialist and generalist could be important in natural ecosystems. For example during diatom blooms, laminarin, an abundant and important carbon source for marine Bacteroidetes, enables them to maintain a large population size and owing to their large PUL repertoire, they can consume other, more complex substrates (Teeling et al., 2012; Krüger et al., 2019; Becker et al., 2020; Francis et al., 2021). In contrast to an 10-fold increase in overall cell numbers during a diatom bloom, potential degraders of fucoidan remain at low abundances in the community and consequently, fucoidan appeared recalcitrant over the course of the bloom (Vidal-Melgosa et al., 2021). A possible explanation is that known fucoidan degraders are substrate specialists and do not grow on labile substrates such as laminarin and their cell numbers might not reach the critical threshold (Sichert et al., 2020). Together this suggests recalcitrance can arise from the low population sizes of specialized degraders, which are limited to reach sufficiently high cell numbers due to their need to specialize on recalcitrant substrates, yet it is an open question if these findings are applicable to other polysaccharides or ecosystems.

## Conclusion

Polysaccharides are the most chemically complex carbon sources available for microorganisms like bacteria, and their enzymatic breakdown and subsequent recycling is key across all ecosystems, from human guts to the ocean. To break down these macromolecules, microorganisms have evolved some of the most sophisticated molecular pathways known, encompassing hundreds of CAZymes that must act in concert. The mapping of substrates to their pathways is an ongoing endeavor which should provide valuable insights into how polysaccharides mediate the coevolution of plants or algae and polysaccharide degrading bacteria. However, the selective pressures operating on CAZyme repertoires may be highly variable, which triggers non-trivial evolutionary dynamics. For example, during early stages of marine particle colonization, there may be a pressure to maintain or expand the repertoire of enzymes, whereas at late stages, organisms may be pressed to not express costly enzymes and instead rely on breakdown products released by other organisms' enzymes. These fluctuations of the selective pressure are perhaps the reason why CAZyme repertoires evolve rapidly, with frequent gene loss and gain constantly shaping the ability of the organisms to degrade polysaccharides.

In contrast to the abundance of sequenced genomes, there is an enormous deficit of phenotypic data for microbes, precluding the development of quantitative theory. This asymmetry between the abundance of genomic and phenotypic data is certainly present in the case of polysaccharide degraders, despite the fact that we have very good hypotheses about which traits matter for ecological success of degraders in the environment. We claim that this is a major problem in the field and that a systematic measurement of key degrader traits, such as the affinity to monomers or enzyme excretion rates, as well as their trait-trait correlations, would be key to help us understand ecological patterns in terms of physiology. This is an important challenge that may require the development of reliable methods to perform physiological measurements in high-throughput. A second, but related problem is the fact that many of these important phenotypes can be plastic, as they are controlled by regulatory programs that respond to chemical cues.

One of the key corollaries to the extracellular breakdown of polysaccharides is the emergence of non-linear growth kinetics, in particular positive density dependences that can be interpreted as “cooperative growth.” In such cases, even if the polysaccharide is intrinsically degradable, it may appear effectively recalcitrant if cell densities are below the critical threshold required to allow the population to grow. This is an important message that tells us that, in order to understand and predict the residence time of polysaccharides in the environment, we need to consider both chemical aspects, such as the decorations that make a polysaccharide harder to degrade, as well as the emergent population dynamics that affect the ability of microorganisms to grow on the substrate.

## Data Availability Statement

The original contributions presented in the study are included in the article/supplementary material, further inquiries can be directed to the corresponding author/s.

## Author Contributions

Both authors listed have made a substantial, direct and intellectual contribution to the work, and approved it for publication.

## Funding

This work was supported by the Simons Collaboration: Principles of Microbial Ecosystems (PriME) Award Number 542395.

## Conflict of Interest

The authors declare that the research was conducted in the absence of any commercial or financial relationships that could be construed as a potential conflict of interest.

## Publisher's Note

All claims expressed in this article are solely those of the authors and do not necessarily represent those of their affiliated organizations, or those of the publisher, the editors and the reviewers. Any product that may be evaluated in this article, or claim that may be made by its manufacturer, is not guaranteed or endorsed by the publisher.
